# Networks uncover hidden lexical borrowing in Indo-European language evolution

**DOI:** 10.1098/rspb.2010.1917

**Published:** 2010-11-24

**Authors:** Shijulal Nelson-Sathi, Johann-Mattis List, Hans Geisler, Heiner Fangerau, Russell D. Gray, William Martin, Tal Dagan

**Affiliations:** 1 Institute of Botany III, Heinrich-Heine University Düsseldorf, Germany; 2 Faculty of Philosophy, Heinrich-Heine University Düsseldorf, Germany; 3 Institute of the History, Philosophy and Ethics of Medicine, Ulm University, Germany; 4 Department of Psychology, University of Auckland, Auckland 1142, New Zealand

**Keywords:** community structure, lateral transfer, phylogenetics

## Abstract

Language evolution is traditionally described in terms of family trees with ancestral languages splitting into descendent languages. However, it has long been recognized that language evolution also entails horizontal components, most commonly through lexical borrowing. For example, the English language was heavily influenced by Old Norse and Old French; eight per cent of its basic vocabulary is borrowed. Borrowing is a distinctly non-tree-like process—akin to horizontal gene transfer in genome evolution—that cannot be recovered by phylogenetic trees. Here, we infer the frequency of hidden borrowing among 2346 cognates (etymologically related words) of basic vocabulary distributed across 84 Indo-European languages. The dataset includes 124 (5%) known borrowings. Applying the uniformitarian principle to inventory dynamics in past and present basic vocabularies, we find that 1373 (61%) of the cognates have been affected by borrowing during their history. Our approach correctly identified 117 (94%) known borrowings. Reconstructed phylogenetic networks that capture both vertical and horizontal components of evolutionary history reveal that, on average, eight per cent of the words of basic vocabulary in each Indo-European language were involved in borrowing during evolution. Basic vocabulary is often assumed to be relatively resistant to borrowing. Our results indicate that the impact of borrowing is far more widespread than previously thought.

## 1. Introduction

Genome evolution and language evolution have a lot in common. Both processes entail evolving elements—genes or words—that are inherited from ancestors to their descendants. The parallels between biological and linguistic evolution were evident both to Charles Darwin, who briefly addressed the topic of language evolution in *The origin of species* [1], and to the linguist August Schleicher, who in an open letter to Ernst Haeckel discussed the similarities between language classification and species evolution [2]. Computational methods that are currently used to reconstruct genome phylogenies can also be used to reconstruct evolutionary trees of languages [3,4]. However, approaches to language phylogeny that are based on bifurcating trees recover vertical inheritance only [3,5–7], neglecting the horizontal component of language evolution (borrowing). Horizontal interactions during language evolution can range from the exchange of just a few words to deep interference [8]. In previous investigations, which focused only on the component of language evolution that is described by a bifurcating tree [3,5–7], the extent of borrowing might therefore have been overlooked.

Lexical borrowing is the transfer of a word from a donor language to a recipient language as a result of a certain kind of contact between the speakers of the two languages [9]. This is one of the most common types of interaction between languages. Lexical borrowing can be reciprocal or unidirectional, and occurs at variable rates during evolution. Factors affecting the rate of lexical borrowing during evolution include the intensity of contact between the speakers of the respective languages, the genetic or typological closeness of the languages (which facilitates the inclusion of foreign words), the amount of bi- or multi-lingual speakers in the respective linguistic communities, or a combination thereof [10,11]. For example, English has been heavily influenced throughout its history by different languages such as Old Norse and Old French [12], it has been estimated that 8 per cent of its basic vocabulary is borrowed from those languages [13]. Icelandic, on the other hand, has preserved most of its original words [14].

**Figure 1.**
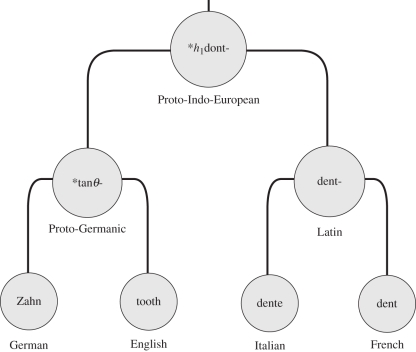
Etymological reconstruction of the concept tooth. The English and German word forms have descended from the Proto-Germanic ancestor [52]. The Italian and French words are descendants of Latin, and the Proto-Germanic and Latin forms stem from Proto-Indo-European [43,53].

A key part of inferences in historical linguistics is the identification of cognate sets. These are sets of words from different languages that are etymologically related. The words in a cognate set are derived from a single common ancestral form that was present in an ancestral language. Cognate judgement is an arduous enterprise since it includes the complete evolutionary reconstruction of all words in the sampled languages for a certain concept. Historical linguists usually make use of an in-depth analysis of structural resemblances between the word forms, looking for sound correspondences in specific environments. The identification of a cognate is thus much more than just a hunt for resemblant forms or ‘lookalikes’. Only a set of words that have regular sound correspondences provide good evidence for genealogical relatedness and thus only these words can be grouped into a single cognate set (COG). For example, the concept ‘tooth’ has a cognate set that unites English *tooth*, German *Zahn*, Italian *dente* and French *dent* as etymologically related (figure 1). However, similar word forms can arise not only by inheritance, but also by lexical borrowing. Unfortunately, the further we go back in time, the more difficult it becomes to distinguish inheritance from transfer, and reconstructed COGs may include hidden borrowing events that are erroneously coded as vertical inheritance.

Lexical borrowing is a non-tree-like evolutionary event that cannot be reconstructed using phylogenetic trees that are common in evolutionary biology [15,16]. Linguists have long been aware of the problems that borrowing introduces. At about the same time that Darwin suggested the tree metaphor for the evolution of species in 1859 [1], August Schleicher introduced the family tree to linguistics [17]. Few years later, his model was rejected by several scholars arguing against the use of a simple tree model to describe the evolution of languages, which they noted to be reticulated by nature [18,19]. Other non-tree-like models were proposed by linguists to study language evolution—including waves [18,20] and networks [21]—but they lacked either quantitative parameters, historical dimensions or both. At the other extreme, quantitative estimates for language divergence lacked an explicit model to explain language relatedness [22,23]. Apart from some sporadic attempts to visualize language evolution of specific words by a combination of a bifurcating family tree with the non-tree-like component superimposed on it [24], linguists have, for lack of better alternatives, largely stuck to the tree model, while emphasizing its inadequacies.

Phylogenetic methods that were developed to take into account horizontal transfer of genes during microbial evolution offer an alternative model for the horizontal aspects of language evolution. Recent years have witnessed several applications of reticulated trees and split networks to language evolution [25–28], yet none of these have either specifically uncovered borrowing events or delivered an estimate for the borrowing frequency during language evolution. Here, we apply phylogenetic networks to recover the frequency of hidden borrowings during the evolution of Indo-European languages using the criterion of word inventory dynamics over time, proposing a general model for language evolution that includes both vertical and horizontal components of word transfer during evolution.

## 2. Methods

### (a) Data

Here, we used two publicly available cognate datasets: Dyen [29] and Tower of Babel (ToB) [30]. For the analysis, all COGs in both datasets are converted into a binary presence/absence pattern (PAP). A PAP within the Dyen dataset includes 84 digits; if a cognate set includes one or more words from language *i*, then digit *x*_*i*_ in its corresponding pattern is ‘1’; otherwise, it is ‘0’. The same conversion method is used for the ToB dataset where the PAPs include 73 digits.

### (b) Shared COGs network

The number of shared COGs between each language pair is calculated as the number of cognate sets in which both languages are present. A division of the network into modules is based on maximizing a modularity function defined as the number of edges within a community minus the expected number of edges [31]. Initially, an optimal division into two components is found by maximizing this function over all possible divisions by using spectral optimization, which is based on the leading eigenvector of the matching modularity matrix. To further subdivide the network into more than two modules, additional subdivisions are made, each time comparing the contribution of the new subdivision with the general modularity score of the entire network. This process is carried out until there are no additional subdivisions that will increase the modularity of the network as a whole [31].

### (c) Reference trees

Language trees were inferred by a Bayesian approach using MrBayes [32] as detailed by Gray & Atkinson [3]. In addition, neighbour-joining (NJ) trees [33] were reconstructed from Hamming distances using SplitsTree [34]. A reference tree with English internal to the Germanic clade was produced manually from the Bayesian tree. A randomized reference tree for the Dyen dataset was produced by randomizing the language names in the Bayesian reference tree. Trees are available in Newick format at http://www.molevol.de/resources.

### (d) Borrowing models and the minimal lateral network

In the loss-only (LO) model, all COGs are assumed to have originated at the root of the reference tree. The loss events for each COG are estimated by using a binary recursive PERL algorithm that scans the reference tree and infers the minimum number of losses [35]. When a COG is absent in a whole clade, a single loss event is inferred in the common ancestor of that clade. In the single-origin (SO) model, each cognate is assumed to have originated at its first occurrence on the reference tree. A binary recursive algorithm scans the reference tree from root to tips to identify the first ancestral node that is the common ancestor of all cognate ‘present’ cases.

In the BOR1 model, each cognate is allowed to have two word origins, where one is a borrowing. A preliminary origin is inferred as in the SO model, followed by researching for a cognate origin in each of the two clades branching from the preliminary origin node. If the hypothetical taxonomic unit that was inferred as the preliminary origin has no cognate ‘absent’ descendants, the cognate is inferred to have an SO. Once the nodes of the two origins are set, losses are inferred as in the LO model.

We tested additional models allowing four, eight and 16 origins, where one is an origin, and the rest are borrowings. These are implemented in the same way as in the BOR1 model, except that the origin search is iterated. For example, a search for origins under the BOR3 model entails (i) a search for a preliminary origin (as in the SO model), (ii) a search for the next origin in descendants (as in the BOR1 model) and, (iii) for each next origin, another search. If an origin has no cognate-absent descendants, the number of origins inferred is smaller than the maximum allowed. Ancestral vocabulary size at a certain internal node is inferred as the total COG origins that were inferred to occur at that node. The distributions of ancestral and modern vocabulary sizes were compared by using the Wilcoxon non-parametric test [36].

The minimal lateral network (MLN) [37] is calculated for each dataset by the allowance model that was statistically accepted by the test described above. The MLN comprises the reference tree, with additional information of the vocabulary size in all internal nodes. Lateral cognate sharing among internal and external nodes is summarized in a 167 × 167 matrix that includes all tree nodes, where *a*_*ij*_ = *a*_*ji*_ = number of laterally shared COGs between nodes *i* and *j*. The MLN is then depicted by an in-house script using Matlab.

## 3. Results and discussion

### (a) Community structure in the network of shared cognate sets

For the study of evolution by borrowing, we analysed two independent, publicly available collections of cognate sets from Indo-European languages. Both datasets comprise words from individual languages or dialects corresponding to concepts that are included in Swadesh lists [38]. Basic concepts are expressed by simple words rather than compounds or phrases and contain names for body parts, pronouns, common verbs and numerals, but exclude technological words and words related to specific ecologies or habitats. Words expressing basic concepts are supposed to exist in all languages and thus may serve as a *tertium comparationis* for language comparison [39]. Moreover, basic concepts are rarely replaced by other words, either through external (lexical borrowing) or internal factors (semantic shift) [13,16].

The Dyen dataset [29] includes word forms for 84 languages (including Greek, Armenian, Celtic, Romance, Germanic, Slavic, Albanian and Indo-Iranian languages) corresponding to 200 basic vocabulary concepts [39] sorted into 2346 COGs [3]. While obvious borrowings were excluded in the original Dyen dataset [29], we used an edited version where 124 marked borrowings are coded into their respective COGs [25]. Detailed reinspection of Romance cognates revealed an additional six hidden borrowings [40] (electronic supplementary material, table S1).

The second dataset is based on etymological dictionaries and Swadesh lists published by the ToB project [30]. It is based on word forms for 110 basic vocabulary items for a total of 98 languages from which we extracted 73 contemporary ones, including languages from the Celtic, Romance, Germanic, Slavic, Albanian and Indo-Iranian branches of Indo-European, sorted into 722 COGs. Detectable borrowings were excluded in the original database; however, a recent detailed screening revealed five undetected borrowings within Romance languages [40].

A network analysis of the distribution of cognate word forms across Indo-European languages should provide new insights into the frequency and distribution of borrowing in Indo-European language history. Networks are mathematical structures used to model pairwise relations between entities. The entities are called vertices and they are linked by edges that represent the connections or interactions between the vertices. A network of *N* vertices can be fully defined by the matrix *A =* [*a*_*ij*_]_*N* *_ _*N*_, with *a*_*ij*_ = *a*_*ji*_ ≠ 0 if a link exists between nodes *i* and *j*, and *a*_*ij*_ = *a*_*ji*_ = 0 otherwise. In the study of Indo-European languages, each language is represented by a vertex, *i*, whereas the elements of the matrix, *A*, correspond to the number of shared cognate sets between language pairs, *a*_*ji*_. Cognate sharing can result either from vertical inheritance or from borrowing.

**Figure 2.**
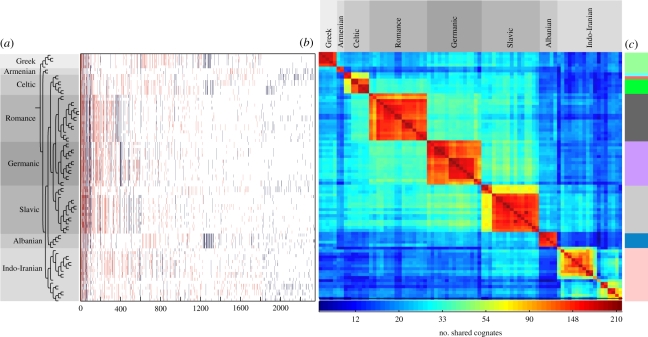
Modules in the shared COGs network. (*a*) A graphic representation of cognate PAPs. Languages are sorted by their order on the reference phylogenetic tree [3]. COGs are sorted by their size in ascending order. A presence case of a certain COG in a certain language is coloured in blue if the COG pattern is congruent with the tree branching patterns and red otherwise. (*b*) A matrix representation of the shared COGs network in Indo-European languages. Cells in the matrix are edges in the network. Edges are colour-coded by the frequency of shared cognate according to the colour bar at the bottom. The languages in the matrix are sorted by order of appearance in the phylogenetic tree on the left. (*c*) Modules within the shared COGs network. Languages included in the same module are coloured in the same colour.

For network reconstruction, cognate sets were converted into a binary format of PAPs for each COG in each language [3]. For the 2346 COGs in the Dyen dataset [29], 1169 different PAPs were observed, of which 942 (80%) are unique and 227 are recurring (figure 2*a*). Closely related languages typically share the most frequent PAPs. For example, Panjabi and Lahnda, two Indian languages, share 78 cognates that are unique to both languages. The ToB dataset includes 532 different PAPs, none of which are unique (electronic supplementary material, figure S1). The frequency of shared COGs among languages in the main branches uncovers components of both inheritance and borrowing.

The binary PAPs of the Dyen COGs are readily assorted into an 84 × 84 matrix representation of the cognate-sharing network that consists of vertices (languages) connected by edges (shared cognates), the edge weights are the number of shared cognates per vertex pair. There are 3486 edges in the network, all vertices of which are connected, thereby forming a ‘clique’ in network terms (figure 2*b*). Some groups of languages are more strongly interconnected among themselves than with others in the cognate-sharing network, thereby forming communities.

We examined the community structure in the network by division into modules [31,41]. Modules correspond to ‘natural’ groups within a network, that is, groups of vertices that are more highly connected to each other than they are to other vertex sets. With only two exceptions, the nine modules calculated within the cognate-sharing network correspond exactly to the main branches of Indo-European languages. One exception concerns the Armenian dialects Adapazar (*Armenian List* in Dyen dataset [42]) and eastern modern Armenian (*Armenian Mod* in Dyen dataset [42]), which are grouped with the Greek languages into one module. This is because Armenian shares significantly (*p* ≪ 0.01, using the Wilcoxon test) more cognates with the Greek languages (30 ± 2, *n* = 5) than with the other languages (22 ± 3, *n* = 79). This module has been independently recognized by linguists [43]. The other exception is the split of both Irish dialects from Celtic (figure 2*c*). The same network-based analysis of the ToB dataset yields only four modules: (i) Slavic and Albanian; (ii) Armenian, Greek, Celtic, Germanic and Romance; (iii) Indo; and (iv) Iranian (electronic supplementary material, figure S2).

Language communities that do not correspond to monophyletic clades in the tree are the result of patchy COG distributions that could not be reconciled with the phylogenetic tree. For example, Romani, which branches with Indo-Iranian languages, shares 25 COGs with Modern Greek, such as the COGs for ‘flower’ (Modern Greek: *λ*ου*λ*ού*δι* (*louloudi*); Romani: *lulugi*) and ‘because’ (Modern Greek: *επειδ*ή (*epeide*); Romani: *epidhi*). Since the Romani dialect in the Dyen dataset [29] is a variety spoken in Greece [42], these are probably borrowed from Greek to Romani.

### (b) Borrowing frequency during Indo-European language evolution

In the Dyen dataset, there are 1391 (59%) patchily distributed PAPs that are incongruent with the tree branching pattern (figure 2*a*). In principle, such patchy COG distributions could arise solely through independent parallel evolution, through vertical inheritance from the common ancestor of all languages and differential loss of lexica during language evolution, or via lexical borrowing among languages. The first possibility seems sufficiently unlikely as to exclude *a priori*. There is no clear estimation for the frequency of parallel evolution during language evolution, but we can assume that it is rather rare and cannot, therefore, be used to explain the distribution pattern of all patchy COGs. If we invoke the second scenario to explain all COGs of patchy distribution, then the result is a common ancestral language that includes each and every COG existing in contemporary languages. In order to entertain such a claim, one would have to assume that the proto-language employed many different, but redundant, words for the same basic concepts, far more than every known contemporary language. This runs contrary to uniformitarianism, a key principle in historical sciences such as geology, biology and linguistics, which states that processes in the past should not be assumed to differ fundamentally from those observed today [44,45]. Hence, if ancient and modern languages were of similar nature, then the number of words that were used to express fundamental concepts (basic vocabulary size) in ancestral languages should be similar to that used in contemporary languages. This principle can be used to infer the minimum amount of lexical borrowing in Indo-European languages that is required in order to bring the distribution of basic vocabulary size in ancestral languages into agreement with that of contemporary languages.

**Figure 3.**
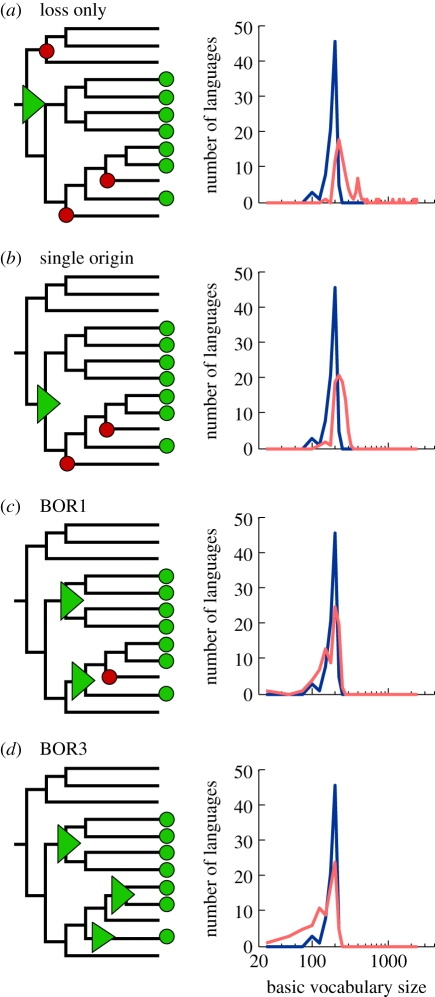
Inference of borrowing frequency by ancestral vocabulary size. (*a*–*d*) Schematic (left) and dynamics of ancestral and contemporary vocabulary size (right) under the different borrowing models. The fraction of interquartile range ((Median_ancestral_ − Median_contemporary_)/IQR_contemporary_) in the different models is as follows. Loss only: 2.92; origin only: 1.93; BOR1: 0.12; BOR3: −0.86. Green triangles, origin; red circles, loss; green circles, word presence; blue line, contemporary languages; red line, ancestral languages.

This network method to address non-tree-like patterns of shared characters requires the use of a reference tree [37]. Here, we use a phylogenetic tree reconstructed by a Bayesian approach [3]. First, we designate an evolutionary scenario that uses vertical inheritance and LO (model), according to which current COG distribution is governed solely by loss. Each ancestral language contains all cognates present in its descendants, and vocabulary size hence becomes progressively larger back through time (figure 3*a*). Note that a loss event applies only to the sample of basic vocabulary and does not mean a loss from the language as a whole. With the Dyen dataset [29] and the reference tree, the common Indo-European ancestor would have had a vocabulary size of 2346 for basic words, expressing 200 basic concepts. This estimate is 11 times larger than the average basic vocabulary size in our sample (*p* = 1.05 × 10^−24^, using the Wilcoxon test). Such large vocabulary sizes are indeed unrealistic, but so is the assumption that new words do not arise during language evolution. In the SO model, we allow new words to arise over time, placing the word origin at the most parsimonious place that is the common ancestor of all COG-present cases (figure 3*b*). This model results in smaller ancestral vocabularies of up to 317 COGs, but these are still significantly larger than the contemporary vocabularies (*p* = 1.65 × 10^−19^, using the Wilcoxon test). The SO model entails an average of three losses per COG (electronic supplementary material, table S2).

Thus, we either have to embrace the untenable assumption that ancestral vocabulary sizes were fundamentally different in the past than they are today or, preferably, we have to allow some amount of borrowing during evolution. We start by allowing only one borrowing event per COG, the BOR1 model. This model allows each COG to have two origins in the reference tree, one of which is by borrowing from any source (figure 3*c*). The result of this model is reduced ancestral vocabularies during the early evolution of languages, and an overall ancestral vocabulary size distribution that is not significantly different from that of contemporary languages (*p* = 0.61, using the Wilcoxon test). Of the total Dyen COGs, 918 (39%) are monophyletic, hence their distribution is readily explained by an SO, while the remaining 1373 (61%) are patchy enough to infer two origins (one borrowing event). This frequency translates to an average rate of 0.6 borrowing events per COG during Indo-European language evolution.

If we allow up to three borrowings per COG (the BOR3 model; figure 3*d*), inferred ancestral vocabulary shrinks towards sizes that are again significantly different from modern ones, but this time are smaller than those of contemporary languages (*p* = 4.43 × 10^−5^, using the Wilcoxon test); that is, too much borrowing and not enough vertical descent are incurred from the standpoint of ancestral vocabulary sizes. Furthermore, under the BOR3 model, the average number of inferred word losses per COG is less than 1. But loss of COGs within basic vocabulary occurs quite frequently in language evolution [7], hence the BOR3 model is also unrealistic in that sense. Additional models allowing up to 15 borrowings per COG result in even smaller ancestral vocabulary sizes (electronic supplementary material, figure S3). Hence, ancestral basic vocabulary sizes demand borrowings to keep them realistically small, but too much borrowing makes them unrealistically small.

Testing the present evolutionary models with the help of a reference tree that is inferred from the same data might bias the inference of origin and loss events. However, using the Bayesian approach to reconstruct the tree yields the majority signal in the data. If the majority of COGs evolve mainly by vertical inheritance, then the tree is expected to be a reliable representation of the language phylogeny [46]. High frequency of borrowing events may mask the vertical signal and lead to less reliable reconstruction. To test the robustness of our borrowing frequency estimates, we repeated our analysis using various reference trees. Use of an alternative phylogenetic tree reconstructed by NJ [33] results in the same BOR1 model (*p* = 0.7, using the Wilcoxon test; electronic supplementary material, figure S3). In both reference trees, English is basal to the Germanic clade. However, this position is debated among linguists, and traditional classifications put English inside that clade [12,47]. To test the influence of the English position within the tree on our borrowing assessment, we tested all models using a reference tree with English in an internal position. Using that reference tree also yielded the BOR1 model (*p* = 0.78, using the Wilcoxon test), with all other models rejected (*α* = 0.05). Using a random phylogenetic tree eliminates all patterns of vertically inherited COGs and accordingly results in the BOR15 model (*p* = 0.16, using the Wilcoxon test; electronic supplementary material, figure S4).

Performing the same tests on the ToB dataset yielded higher borrowing frequencies, with BOR3 being the only statistically accepted model (*p* = 0.59, using the Wilcoxon test; electronic supplementary material, figure S5). Inference by this model results in 155 COGs of SO, 181 COGs of two origins, 307 COGs of three origins and 79 COGs of four origins. Hence, in 567 (79%) of the 722 COGs, we detected one or more borrowing event. The average rate of borrowing events per COG during language evolution in the ToB dataset is 1.4 (electronic supplementary material, table S2). The higher borrowing rate inferred for the ToB dataset in comparison to the Dyen dataset might have to do with differences in their reconstruction. The cognate judgements in ToB are based on a deeper etymological reconstruction in comparison to the Dyen dataset. This results in more words that are distributed over fewer cognate sets, which leads to patchy COG distribution patterns that are frequently incongruent with the phylogenetic tree.

The sample of languages is crucial for the distinction between COG origin by birth or borrowing because what may seem to be a word birth within a given sample of languages in our data could in fact be a borrowing event from a non-sampled language. How severe is the effect of external borrowing on our results? If we assume the extreme case, for example, that all COGs in the dataset originated by borrowing from external languages, then we have to add one borrowing event to the average rate for each COG. In that case, the average borrowing rate would increase from 0.6 to 1.6 events per COG using the Dyen dataset. However, this extreme scenario is unlikely because it entails the assumption that the Indo-European groups sampled here lacked the wherewithal to invent even one new COG. Nonetheless, external borrowing has almost certainly had an effect on these data. Although we currently lack a dataset that would allow us to quantify the rate of external borrowing, if we assume that it is similar to the internal borrowing rate within our sample, the overall borrowing rate would be double our current estimate. Again we stress that the borrowing frequency inferred from the present sample of languages using our method delivers a minimum value (a conservative lower bound).

Another aspect of the data sample used in our analysis is the collection of cognates. Here, we study the dynamics of vocabulary size during evolution through the proxy of basic vocabulary (i.e. the Swadesh list). However, origin and loss of words in the COGs sample can occur by semantic shift where the word is present in the language but absent from the sample. It is possible that different meaning collections evolve under regimens different from the ones described here. Application of similar methods to study vocabulary size dynamics over time using different cognate datasets will help to clarify this issue.

Notwithstanding certain amounts of cognate misjudgements and parallel evolution [48] resulting in tree-incompatible COG distributions, our inference uncovers abundant, and hitherto unrecognized, borrowing during the evolution of the Indo-European languages.

Scholars usually agree that nouns are more easily borrowed than verbs [49]. When classified according to the English gloss, the Dyen dataset includes 887 (53%) cognate sets corresponding to nouns within basic vocabulary and 766 (46%) cognate sets corresponding to verbs. A total of 503 (53%) nominal cognate sets and 450 (47%) verbal cognate sets were identified as including hidden borrowing events. A comparison of these frequencies shows that there is no significant difference in borrowing frequencies between nouns and verbs (*p* = 0.4, using the *G*-test).

### (c) Minimal lateral networks of Indo-European languages

**Figure 4.**
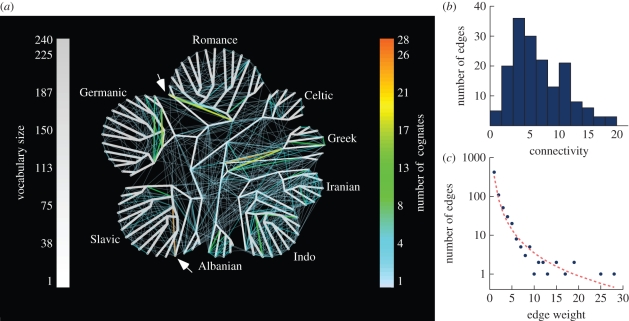
The MLN of Indo-European languages. (*a*) An MLN for 84 contemporary languages reconstructed under the BOR1 model. Vertical edges are indicated in grey, with both the width and the shading of the edge shown proportional to the number of inferred vertically inherited COGs along the edge (see the scale). The lateral network is indicated by edges that do not map onto the vertical component, with the number of cognates per edge indicated in colour (see the scale). Lateral edges that link ancestral nodes represent laterally shared COGs among the descendent languages of the connected nodes, whose distribution pattern could not be explained by origin and LO under the ancestral vocabulary size constraint. The two heaviest edges of Slovene (Slavic) and Romanian (Romance) are marked by an arrow. (*b*) Distribution of connectivity, the number of one-edge-distanced neighbours for each vertex, in the network. (*c*) Frequency distribution of edge weight in the lateral component of the network.

COG distributions that do not map exactly onto the phylogenetic tree, with borrowing constrained by ancestral vocabulary size only, constitute the MLN [37]. The MLN reconstructed from the Dyen dataset consists of 167 vertices, of which 84 are contemporary and 83 are ancestral languages (internal nodes in the reference tree). The vertices are interconnected either by the branches of the reference tree, representing vertical inheritance, or by lateral edges, representing horizontal transfer (figure 4*a*).

The internal and external vertices in the MLN for the broad sample of COGs are linked by 666 lateral edges. The connectivity (number of edges per vertex) within the MLN ranges between 0 and 21 edges per language, with a median of 7 (figure 4*b*). The most highly connected node is Ossetic (21 edges), an east Iranian language, which is connected with Indo-Iranian, Greek and Slavic languages. Lateral edges connected to external nodes correspond to comparatively recent borrowing events. On average 8 ± 7% COGs per language are involved in recent borrowing (electronic supplementary material, table S3). This result suggests that English, at 8 per cent borrowing rate [13], is not exceptional; it is merely the most studied language. The clustering coefficient of the MLN is 0.22, and the mean shortest path is 3.128 edges. Combined with the high level of clustering, this means that the MLN forms a small-world network.

The edge weight distribution within the MLN is characterized by a majority of small edge weights. Of the total edges, 422 (63%) are of a single laterally shared COG, while edges of multiple COGs are rare (figure 4*c*). The two heaviest lateral edges include an edge between Slovene and the remaining Slavic languages (28 COGs), and an edge between Romanian and the remaining Romance languages (19 COGs). These lateral edges uncover a certain kind of language change that results from the same evolutionary process. Both Slovene and Romanian, being heavily influenced by neighbouring languages, underwent a process of linguistic revival starting from the early 19th century, in which the original traits that had been lost during long periods of contact were artificially reintroduced into the languages by the speakers in order to bring them back to a stage of earlier ‘purity’ [50,51]. Before the 19th century, Slovene comprised several dialects spoken in the Alpine provinces of the Austrian Empire, which were dominated by German and Italian. Romanian, on the other hand, was heavily influenced by neighbouring Slavic and Greek varieties, with which it formed the so-called Balkan *Sprachbund*. Along with the nationalist movements in Europe starting from the end of the 18th century, both languages were successively ‘purified’ by replacing the loanwords of non-Slavic or non-Romance origin with ‘native’ words from Slavic or Romance languages, respectively [50,51]. This process is somewhat different from the process of borrowing as it was defined in the beginning of this paper. It nonetheless illustrates additional horizontal complexities in the processes of language evolution that are readily detected in the MLN.

The comparison between the edges reconstructed using the two reference trees that differ in their English position supplies a few interesting observations regarding the applicability of our approach to detect borrowing events. While both reference trees yielded the same borrowing model (i.e. the same overall borrowing rates), there are 23 lateral edges connecting to English in the basal position and only 15 lateral edges connecting to English in the internal position. A closer inspection of the COGs in which the lateral edges connecting to English were detected revealed that seven of the eight COGs detected as borrowings in the basal position could not be verified as borrowings by traditional historical linguistics. Thus, using different reference trees with the same COG distribution patterns does not much affect the resulting borrowing model, but it may increase the accuracy of concrete predictions made by this approach (see electronic supplementary material, table S4 for detailed etymological reconstruction of the COGs). Consequently, the borrowing inference accuracy in our approach is expected to increase with the accuracy of the reference tree.

The MLN inferred from the ToB dataset shows similar network characteristics, with the ancestors of Indian and Iranian clades found also as highly connected nodes and a majority (676; 76%) of single laterally shared COGs (electronic supplementary material, figure S6).

Of the total 666 edges in the MLN reconstructed for the Dyen dataset, 148 (22%) edges connect between two external nodes—that is, between two contemporary languages. The 301 (45%) edges that connect between an internal node and an external node represent COGs that are shared between a group and an outlier. The 217 (33%) edges that connect between two internal nodes represent COGs that are common to two different groups, yet their distribution pattern could not be explained by vertical inheritance alone under the vocabulary size criterion. As a control to see whether our method is inferring spurious borrowing, we examined the edges within cognates that included the 124 reinserted borrowing events. In seven cognates, the algorithm detected no borrowings, while in all other 117 (94%) cognates a borrowing event was inferred. In 59 (48%), the reinserted borrowing language was inferred as an external node. In the remaining 58 (47%), reinserted borrowing languages were inferred within descendants of an internal node (table 1).

**Table 1.** Reconstructed borrowing events. The origin node that includes the reinserted borrowing is shaded in light grey.

edge type	origin node	number of reinserted borrowings
external–external	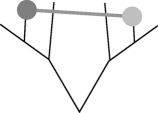	1
external–internal	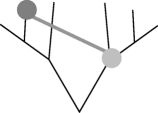	18
	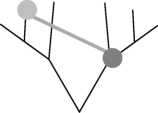	58
internal–internal	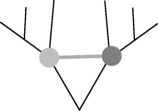	40

The data can address the issue of whether words are exchanged more frequently within than between main branches of Indo-European. We can compare the probability of a certain language to be laterally connected with languages that are either from the same main branch or from different main branches of the Indo-European languages. With the exception of the Armenian branch, the probability for a lateral edge within the branch (internal edge) is considerably higher than between branches (external edge). Furthermore, lateral edge weights are significantly larger in internal lateral edges than in external lateral edges (table 2). Hence, lexical borrowing in Indo-European languages is much more frequent among languages within the same branch in comparison to languages from different branches. This provides new evidence for the existence of certain cultural barriers to lexical borrowing during language evolution [10].

**Table 2.** Lateral edge (LE) frequencies between and within groups in the MLN.

		normalized borrowing		median LE weight^b^		*H*_0_:LE_int_ ≤ LE_ext_ frequency^c,d^
group	*n*^a^	int	ext	int	ext	*p*-value
Greek	9	1.22	0.25	2	1	<0.05
Armenian	3	0	0.17	0	1	n.a.
Celtic	13	1.61	0.29	2	1	≪0.05
Romance	31	2.45	0.36	1	1	≪0.05
Germanic	29	2.37	0.44	1	1	≪0.05
Slavic	31	2.35	0.64	1	1	≪0.05
Albanian	9	1.55	0.18	4	1	≪0.05
Indic	21	3.33	0.68	2	1	≪0.05
Iranian	14	2.35	0.75	2	1	≪0.05

^a^Number of languages within group.

^b^Range of median number of COGs per lateral edge.

^c^One-side Kolmogorov–Smirnov test for lateral edge distribution.

^d^For internal edges (int), number of internal edges per number of nodes within the group; for external edges (ext), number of external edges per number of nodes outside the group.
